# The Prevalence of Musculoskeletal Pain (MSP) Among Orthopedic Surgeons and Residents in Saudi Arabia's Eastern Area

**DOI:** 10.7759/cureus.39246

**Published:** 2023-05-19

**Authors:** Fahad A Al Mulhim, Hajer E AlSaif, Mohammad H Alatiyah, Mohammed H Alrashed, Abdulmohsen A Balghunaim, Adnan S Almajed

**Affiliations:** 1 Orthopaedic Surgery, King Fahad Hospital Hofuf, Al-Ahssa, SAU; 2 College of Medicine, King Faisal University, Al-Ahssa, SAU

**Keywords:** saudi arabia, surgeons, pain, musculoskeletal, prevalence

## Abstract

Background: Orthopedic surgery is a tiring specialty both physically and mentally. Surgeons tend to hold strenuous postures for long periods of time. Orthopedic surgery residents are affected just as much as their seniors by the difficult ergonomics. More care should be aimed toward healthcare professionals to improve patient outcomes and decrease the burden on our surgeons. The goal of this study is to pinpoint the areas of musculoskeletal pain among orthopedic surgery physicians and residents and its prevalence in the eastern province of Saudi Arabia.

Methods: A cross-sectional study was conducted in the Eastern region of Saudi Arabia. A simple random selection of 103 male and female orthopedic surgery residents from Saudi Commission for Health Specialties accredited hospitals was enrolled in the study. Residents enrolled from the first to fifth year. Data were collected using a self-administered online questionnaire based on the musculoskeletal Nordic questionnaire activated in 2022-2023.

Results: Out of 103, a total of 83 completed the survey. The majority (49.9%) were junior residents from residency year (R) 1-R3 and exactly 52 (62.7%) residents were males. The majority of the participants, which were 35 physicians (55.6%), perfume less than six operations as average operations per week, and duration stay in the operating room (OR) per operation there were 29 physicians (46%) stay in the OR for 3-6 h. The most reported sites of pain included lower back pain (46%), followed by neck pain (39.7%) and then upper back pain (30.2%). About 27% of the participants had the pain for more than 6 months, however, only 7 (11.1%) residents seek for medical help.

Considering the associated factors with MSP, smoking, and residency year were significantly associated with having musculoskeletal pain (MSP). The presence of MSK pain among R1 residents represents 89.5%, in comparison with R2 residents Who reported 63.6% and 66.7% among R5 residents. This finding indicates a decrease in MSP among residents over the 5 years of residency programs. Additionally, the majority of the participants with MSP reported being smokers 24 (88.9%), controversy, only three of the participants represent (11.1%) without MSP and smokers.

Conclusions: Musculoskeletal pain is a serious issue that needs to be addressed. The results indicate that the most reported areas of MSP were the low back, neck, and upper back. Only a minority of the participants went to seek medical help. Residents from R1 experienced more MSP than their seniors and this could indicate an adaptive behavior from senior staff. More research should be done on the topic of MSP in order to promote health among caregivers across the kingdom.

## Introduction

 Orthopedic surgeons work in a dangerous field that has infections, radiation, smoking, toxins, excessive noise, musculoskeletal injuries, as well as emotional and psychological problems which are all risks that orthopedists are more likely to experience [1]. It is logical to put the emphasis on patients’ health but it is easy to overlook the healthcare providers’ well being [2]. The practice of orthopedic surgery requires a lot of stamina for long hours of the week [3]. The areas that were mostly reported for musculoskeletal pain by orthopedic surgeons were the neck, lower back, shoulder, and elbow [4]. Among the risk factors for musculoskeletal pain (MSP) is the awkward posture that surgeons tend to hold while operating [5]. The use of loupe magnification and headlight are also reported to cause MSP [6]. Increased age and the number of cases held by the surgeon are also aggravating factors for MSP [2-7]. Even though orthopedic surgery residents do not operate as much as their seniors, they still provide support during these operations which mostly involve tasks that are tiring such as holding extremities for prolonged periods of time [8]. A study mentioned that orthopedic surgery residents encounter the same amount of pain that practicing surgeons experience [9]. The difficulties and injuries that surgeons face can have a profound negative impact physically, mentally, and financially [2-7]. There is a small number of papers that are published regarding the topic of musculoskeletal disorders among orthopedic surgery practitioners and residents [9]. The aim of this study is to quantify the prevalence of MSPs among practicing orthopedic surgery physicians and residents in the eastern province of Saudi Arabia.

## Materials and methods

A quantitative cross-sectional study will be conducted among orthopedic surgery residents. A validated self-report questionnaire will be distributed to all residents enrolled from the 1st to 5th year in the residency program in the Eastern region, Saudi Arabia using Nordic Musculoskeletal Questionnaire. The questionnaire will be activated in 2022-2023 and the access to the survey could be from any electronic device.

Inclusion criteria and exclusion criteria

All the enrolled orthopedic surgery residents from the 1st to 5th year in the orthopedic residency program in the Eastern region in Saudi Arabia. The target participants should be 24 years old or above including both males and females who are currently enrolled in an orthopedic residency program in the Eastern region of Saudi Arabia. Orthopedic surgeon residents enrolled in orthopedic residency programs outside the Eastern region, inside the Eastern region but consultants and specialists or orthopedic surgeon residents who refuse the informed consent are excluded.

Sampling technique and sample size calculation

The data are collected as a simple random selection of 103 orthopedic surgery residents from Saudi Commission for Health Specialties accredited hospitals in the Eastern region using Google Forms through a self-administered online questionnaire. The determinations of the minimum sample size were done using the Richard Geiger Richard-Geiger equation; the marginal error was determined as 5%, and the 95% confidence level is 82.

Data collection procedures

The questionnaire includes gender, age, level/degree, years of experience, weight, height, smoking, exercise, average hours in the operating room, handedness, and the presence of MSP. Additionally, questions related to two validated screening tools are Nordic Musculoskeletal Questionnaire to screen musculoskeletal disorders as well as occupational healthcare practice [10]. The questionnaire analyzes the symptoms crossing nine different anatomic regions which are neck, upper back, lower back, shoulders, elbows, wrists/hands, hips/thighs, knees, and ankles/feet. The survey questionnaire is taken from a previous article that formulated the questions and was reviewed by experts [2].

Participants confidentiality and data privacy are our top priority as informed consent was taken by electronic Google Form survey. No names or personal information will be disclosed avoiding any ethical issue. The ethical clearance was acquired by the Deanship of Scientific Research, King Faisal University before initiating the study (reference No. KFU-REC-2022-APR-EA000567).

Data management and statistical analysis

Data were collected, reviewed, and processed using the Social Sciences Version 21 Statistical Package (SPSS: IBM company, Armonk, NY). All statistical methods used were two-tailed with an alpha level of 0.05, considering significance if the p value is less than or equal to 0.05. The prevalence of MSP was graphed among the study residents. Descriptive analyses were performed by prescribing frequency distributions and percentages of study variables, including resident sociodemographic and work-related data. Also, residents pain site, frequency, severity, and associated operating room (OR) frequency days and hours were tabulated. Cross tabulation analysis was used for showing factors associated with having MSP using Pearson chi-square test for significance and exact probability to notice if there were small frequency distributions and differences.

## Results

Table 1 shows a total of 83 residents were included. An exact of 19 (22.9%) were in their first residency year, 22 (26.5%) in their second residency year, and 28 (33.7%) were at R4 and R5. Resident ages ranged from 25 to 36 years with a mean age of 27.8 ± 2.2 years old. Exact of 52 (62.7%) residents were males and 31 (37.3%) were females. Considering body mass index (BMI), 22 (26.5%) had overweight and 12 (14.5%) were obese. A total of 27 (32.5%) were smokers and 36 (43.3%) exercises regularly. The right hand was the dominant hand among 73 (88%) residents.

**Table 1:** Personal characteristics of study orthopedic residents.

Personal data	No	%
Age in years		
25-26	28	33.7
27-29	37	44.6
30-36	18	21.7
Gender		
Male	52	62.7
Female	31	37.3
Residency year		
R1	19	22.9
R2	22	26.5
R3	14	16.9
R4	19	22.9
R5	9	10.8
Body mass index		
Normal weight	49	59.0
Overweight	22	26.5
Obese	12	14.5
Smoking		
Yes	27	32.5
No	56	67.5
Regular exercises		
Yes	36	43.4
No	47	56.6
Dominant hand		
Right hand	73	88.0
Left hand	10	12.0

Table 2 shows that 35 (55.6%) of the study residents do less than six operations per week, 21 (33.3%) do 6-10 operations per week while 7 (11.1%) do more than 10 operations. An exact of 27 (42.9%) stay in the OR for less than 3 h per operation and 29 (46%) stay for 3-6 h. As for experience years, 23 (27.7%) had at least 1 year of experience, 27 (32.5%) had 2-4 years, and 33 (39.8%) had 5 years or more.

**Table 2:** Work data among orthopedic residents.

Work data	No	%
As an average, how many operations do you do per week?		
< 6 operations	35	55.6
6-10 operations	21	33.3
> 10 operations	7	11.1
As an average, how long do you stay in the OR per operation?		
< 3 h	27	42.9
3-6 h	29	46.0
> 6 h	7	11.1
Years of experience		
0-1	23	27.7
2-4	27	32.5
5+	33	39.8

**Figure 1:**
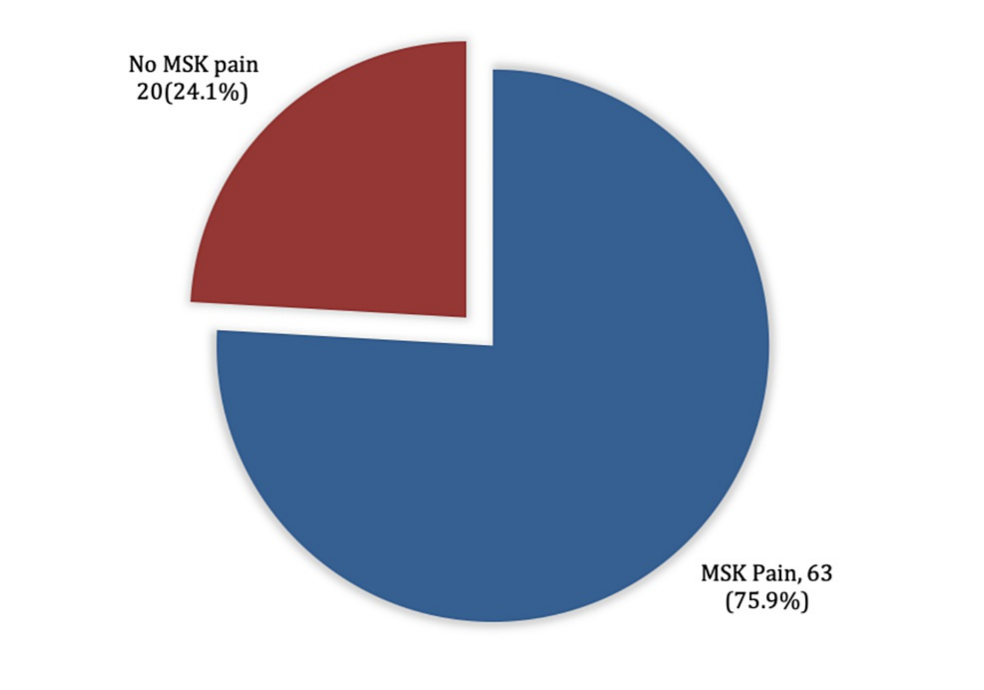
Prevalence of MSK pain. MSK, musculoskeletal

Figure 1 shows the percentage of our population with or without MSP. Exact of 63 (75.9%) complained of MSP.

Table 3 shows clinical characteristics of MSP experienced among orthopedic residents in the eastern province of Saudi Arabia. As for the site of pain, the most reported sites included lower back pain (46%), neck pain (39.7%), upper back pain (30.2%), shoulder pain (26.6%), and knee pain (22.2) while 22.2% had general MSP. The pain was mild among 50.8%, moderate among 46%, but severe among only 3.2%. An exact of 29 (46%) had pain for less than 1 week, 14.3% for 1-4 weeks, and 27% had pain for more than 6 months. An exact of 37 (58.7%) residents had less than five episodes per month. Only 7 (11.1%) seek for medical help and 5 (7.9%) had taken sick leave because of the pain.

**Table 3:** Clinical characteristics of MSP experienced. MSP, musculoskeletal pain

Pain characteristics	No	%
Site of pain		
Lower back pain	29	46.0
Neck pain	25	39.7
Upper back pain	19	30.2
Shoulder pain	18	28.6
General musculoskeletal pain	14	22.2
Knee pain	14	22.2
Hand and wrist pain	4	6.3
Foot and ankle pain	3	4.8
Elbows pain	2	3.2
Others	5	7.9
Severity of pain		
Mild pain (0-4)	32	50.8
Moderate pain (5-7)	29	46.0
Sever pain (8-10)	2	3.2
Duration of pain		
Less than 1 week	29	46.0
1-4 weeks	9	14.3
2-3 months	5	7.9
3-6 months	3	4.8
> 6 months	17	27.0
Frequency of pain attacks (episodes per month)		
< 5 episodes	37	58.7
> 5 episodes	26	41.3
Did you seek medical help?		
Yes	7	11.1
No	56	88.9
Have you taken sick leave because of the pain?		
Yes	5	7.9
No	58	92.1

Table 4 shows factors associated with having MSP among study orthopedic residents. Only smoking and residency years were significantly associated with having MSP. A total of 88.9% of smokers had pain versus 69.6% of non-smokers (p=0.043). Also, MSP was reported among 89.5% of R1 residents compared to 66.7% of R5 residents (p=0.049). 

**Table 4:** Factors associated with having MSP. *p < 0.05 (significant); R, residency year MSP, musculoskeletal pain

Factors	Musculoskeletal pain				p-value
	Yes		No		
	No	%	No	%	
Age in years					0.976
25-26	21	75.0	7	25.0	
27-29	28	75.7	9	24.3	
30-36	14	77.8	4	22.2	
Gender					0.803
Male	39	75.0	13	25.0	
Female	24	77.4	7	22.6	
Body mass index					0.562
Normal weight	38	77.6	11	22.4	
Overweight	15	68.2	7	31.8	
Obese	10	83.3	2	16.7	
Smoking					0.043*
Yes	24	88.9	3	11.1	
No	39	69.6	17	30.4	
Regular exercises					0.386
Yes	29	80.6	7	19.4	
No	34	72.3	13	27.7	
Residency year					0.049*
R1	17	89.5	2	10.5	
R2	14	63.6	8	36.4	
R3	12	85.7	2	14.3	
R4	14	73.7	5	26.3	
R5	6	66.7	3	33.3	
Years of experience					0.708
0-1	17	73.9	6	26.1	
2-4	22	81.5	5	18.5	
5+	24	72.7	9	27.3	

## Discussion

Orthopedic surgeons are exposed to ergonomically challenging postures [11]. These difficult postures have been recognized as risk factors for musculoskeletal disorders in the profession [12-13]. Also, orthopedic surgery mostly needs substantial physical effort to operate and support heavy operative skills [11]. Although very few data have been published about MSP in many orthopedic operations, maintaining neck flexion and elevating the arm and shoulder positions during surgery are known to be major MSP risk factors [14]. Likewise, it has been established that orthopedic surgeons experience a higher rate of procedural pain than many other specialties due to long surgical procedures [15-16]. The current study aimed to assess the prevalence of MSP among orthopedic surgeons and residents in the eastern province of Saudi Arabia. The study showed that more than three-fourths of the residents experienced MSP. Experienced pain was significantly higher among smokers and R1 residents who were mostly not used to spend too long time in OR in contrast to R5 residents who were experienced with procedures. The study also showed that the pain was mild among half of them which was for less than 1 week mainly but one-fourth of them had chronic pain for more than 6 months. Also, more than half of those with MSP experienced less than 5 episodes per month. The most reported pain type included lower back pain, neck pain, upper back pain, shoulder pain, and knee pain while less than one-fifth had general MSP. A study in Saudi Arabia conducted by Aljohani and King Fahad General Hospital revealed similar findings: lower back pain was the most reported, followed by neck pain [17]. Also, about 23% of participants have had MSP for longer than six months, and 9.3% experienced MSP more frequently than five times per week. Another study conducted in Saudi Arabia revealed that 67.0% of the participants reported experiencing MSP. The most commonly reported MSP was in the lower back represented (74.0%), followed by the neck represented (58.2%) [18]. A lower prevalence was reported in Riyadh (67%) and India (50.7%) [7-19]. McQuivey et al. found that almost all residents (97%) complained of MSP [8]. The average pain score of all residents was 3.52/10. The most reported type of pain included lower back (35%), neck (29.7%), and feet (25.7%). Also, Knudsen et al. reported that neck pain was the most frequent among orthopedic surgery residents (59%), followed by lower back (55%), upper back (35%), and shoulders (34%) [9].

The study further investigated the relationship between the effect of MSP and medical needs among the residents. The current study showed that only seven (11.1%) seek for medical help and five (7.9%) had taken sick leave because of the pain. This is explained by that the pain was mild among more than half of them and also it lasted for less than 1 week while few percent experienced chronic pain. Aljohani and King Fahad General Hospital reported that nearly 22.7% and 13.4% of orthopedic residents with MSP seek medical advice once [17]. Among participants, only 22.7% reported that MSP is affecting their life. A study in Riyadh showed that the effect of MSP among surgeons’ work was low as the frequency was less than five days per week in 40% and 41% did not take any sick leave due to MSP during the last year.

Furthermore, only one-fifth of orthopedic surgeons seek medical advice which is lower than the prevalence of orthopedic surgeons in Riyadh [20]. The literature review concluded a positive impact of strength training on MSP. Doing strength exercises provide successful pain if it is performed at least three times per week for at least 20 min, and provide successful pain management [21].

## Conclusions

Our study concluded that evidence of physical MSP is a serious issue that needs to be addressed. The most reported areas of MSP were the low back, neck, and upper back. Only a minority of the respondents went to seek medical help which shows it is not a server. Residents from R1 experienced more MSP than their seniors and this could indicate an adaptive behavior from senior staff. Also, smokers have shown proneness to have MSPs which need to be addressed as a serious issue. More research should be done on the topic of MSP in order to promote health among caregivers across the kingdom.
